# Nurses and policymakers role in preparing adolescents with HIV for self-disclosure in Eswatini

**DOI:** 10.4102/phcfm.v16i1.4332

**Published:** 2024-10-09

**Authors:** Baliwe P. Dlamini, Ntombifikile G. Mtshali

**Affiliations:** 1 College of Health Sciences, School of Nursing and Public Health, University of KwaZulu-Natal, Durban, South Africa

**Keywords:** adolescents, disclosure, Eswatini, nurses, HIV, policymakers

## Abstract

**Background:**

Nurses in Eswatini are best positioned to assist adolescents living with HIV to disclose their status to others. Nonetheless, it is evident that many nurses are not actively involved in the disclosure process.

**Aim:**

The aim of this study was to explore the process of nurses in preparing adolescents for self-disclosure and describe the role of policymakers in enabling adolescents to disclose their HIV status to others.

**Setting:**

The study was conducted in four facilities, one from each of the four regions of the country, after getting ethical clearance from a Public University Higher Degrees Ethics Committee and the Eswatini Health and Human Research Review Board.

**Methods:**

In-depth interviews were conducted on 28 participants: 24 nurses and 4 policymakers. The three steps of open, axial and selective coding were used to analyse data until theoretical saturation was achieved.

**Results:**

Adolescents were assisted to disclose by providing them with HIV information to empower them, encouraged to enrol in teen club because it created a conducive environment for peer-to-peer support, and they were given ongoing psychosocial support to prepare them for self-disclosure. Adolescent HIV management workshops were not routinely done because such training relied on funders.

**Conclusion:**

Nurses are not preparing adolescents satisfactorily to disclose. Prioritising the training of nurses would lead to a remarkable increase in the rate of HIV self-disclosure by Swati adolescents.

**Contribution:**

This study is the first of its kind in Eswatini, and the results will contribute to the review of HIV management guidelines and promote adolescent self-disclosure.

## Introduction

Globally, an estimated 1.8 million adolescents (10–19 years) are living with HIV and about 89% reside in sub-Saharan Africa (SSA).^1^ Disclosing HIV status to children early in their lives is a major challenge encountered by families and healthcare providers.^2^ Delayed disclosure prevents children and adolescents from making right and informed decisions regarding their health and increases their risk of onward transmission of HIV to prospective sexual partners and others.^3^ In numerous countries, HIV is still a stigmatised and sensitive topic and HIV status disclosure remains a critical issue in HIV management and yet a key psychosocial matter confronting individuals living with human immunodeficiency virus (HIV) and acquired immunodeficiency syndrome (AIDS).^4,5^ Stressors that come with living with HIV and AIDS entail making an autonomous decision to disclose status to friends and/or partners, retention in HIV care and adhering to anti-retroviral therapy (ART) to ensure viral suppression.^6^

Healthcare practitioners (HCPs) play a pivotal role in the disclosure process because they offer medical and psychosocial care to children living with HIV and their immediate families.^7^ The clinical, psychological, social and reproductive health needs of adolescents have not been thoroughly examined by practitioners, and efforts to mobilise and advocate for their treatment and support have recently been initiated.^8^ Past studies have focused on the role of HCPs in relation to HIV status disclosure to the adolescent, and as an emerging area of enquiry, no documented study has explored the role of HCPs in supporting adolescents living with HIV (ALHIV) to disclose their status to others.^7,9,10^

Studies that have focused on healthcare workers’ experiences of HIV disclosure to children in SSA have asserted that there is a shortage of training and mentoring to improve their skills in the disclosure process and the absence of standardised disclosure policies and guidelines.^7,11^ Stigma and discrimination against people living with HIV deter them from disclosing their status to others and this intensifies in resource-limited settings because of inadequate number of healthcare providers and limited training in HIV disclosure and counselling.^12^ As such, researchers and policymakers have progressively come to fully comprehend the repercussions of HIV disclosure in prevention, treatment and stigma reduction.^13^

Eswatini has been providing ART for more than a decade, but despite this milestone, there is insufficient data and research that has been carried out to explore the role of nurses in the self-disclosure of status by perinatally infected ALHIV. In Eswatini, nurses who form part of HCPs play a crucial role in providing care to children living with HIV, and this therefore necessitates exploring how they equip adolescents to self-disclose in order to inform the development of practical and contextually relevant guidelines that would lead to an increased disclosure rate by ALHIV.^10^ Therefore, this article aims to explore the process followed by nurses in preparing adolescents for self-disclosure and describe the role of policymakers in enabling adolescents to disclose their HIV status to others.

## Research methods and design

### Research design

A qualitative grounded theory design was used in the study. A grounded theory design is used to gain new insights about an unfamiliar phenomenon and grounded theory is the best-suited qualitative design to explain issues that need profound exploration that may lead to novel discoveries.^14,15^ This approach was considered as appropriate for the study because the role of nurses in preparing adolescents for self-disclosure of HIV status in Eswatini and other countries has not been explored before.

### Research setting

The study was conducted in four selected facilities in Eswatini: three of the facilities are government-owned and one facility is privately owned. Cluster sampling was used to select one facility under each of the four regions of Eswatini: Hhohho, Shiselweni, Lubombo and Manzini to ensure representation in the study. Facilities A and B are located in the rural areas of Hhohho and Shiselweni regions, respectively, and serve a high population of adolescents and adults living with HIV. Facility C is located in the Lubombo region and is a peri-urban site also catering for a high population of adolescents living and adults with HIV. Facility D is an urban and private facility located in the Manzini region and a specialised HIV and AIDS clinic that only provides comprehensive HIV care for all people living with HIV. No other services are rendered in this facility besides HIV and AIDS services. Most of the children who are receiving ART from these facilities are from nearby villages. The facilities chosen were well suited for qualitative research as they enabled the researcher to collect data in natural and context-dependent settings. The settings required the researcher to have a deep understanding of contextual features and how these exert their influence on the way participants ascribe meaning to events shaped by their unique circumstances.^16^

### Participants and recruitment strategy

The study population was 28 participants, made up of 24 nurses and four policymakers involved in the development and implementation of policies that address HIV prevention and treatment needs of adolescents. Purposive sampling, which is a non-probability sampling strategy, was used to recruit participants in this study.^17^ Therefore, participants who have experienced the phenomenon being studied were purposively selected to be part of the study because they were going to provide better insight into the research question to achieve the study’s purpose.^18^

The inclusion criteria to the study for nurses were working in the ART department for a minimum of 1 to 2 years or more years of experience, having been trained in basic integrated management of adolescent and adult illness (IMAI) and nurse-led anti-retroviral therapy initiation in Eswatini (NARTIS). For policymakers, the inclusion criteria were having at least 1 year of experience at the policymaking level with more years of experience preferred. To recruit nurse participants, the Medical Officer or Sister-in-Charge of the facility was approached with a letter requesting to conduct the study in the facility.

After a letter granting permission to conduct the study was issued, the researcher was referred to a focal person at the ART clinic by the Medical Officer or Sister-in-Charge to help the researcher approach prospective participants. From the eligible population, each prospective participant was assigned a number starting from one. The numbers were then drawn until the sample size was considered sufficient. After introducing the study, the focal person identified personnel who met the inclusion criteria for the study and further advised the researcher on the appropriate day to come back to present the study when most staff members would be available. On the agreed date, the afternoon was utilised to present the study to prospective participants because of the low client volume, and they were informed about the purpose of the study, applicable ethics to be upheld like voluntary participation, confidentiality and data collection methods to be used in a room allocated to the researcher for the purpose of introducing the study. Participants were given the information sheet to go through, and afterwards, both the researcher and participants agreed on the date to commence data collection. On the day of data collection, participants first signed the consent forms, and thereafter, data collection commenced. Data saturation and repetition of themes determined the final sample size. To recruit policymaker participants, the researcher first presented the study in their respective offices and went through the information sheet and explained the purpose of the study to them. All policymakers who were recruited are at the government national level and their involvement in HIV policy entails providing technical leadership to harmonise the delivery of HIV and AIDS care and treatment services in line with national policies. Furthermore, they coordinate adolescent health at the national level, working with supporting partners to fund training and build capacity of healthcare workers on reproductive health issues including HIV and AIDS to improve youth friendly services and providing psychosocial support to people living with HIV to promote their health and well-being through liaising with community support groups. The researcher was given a suitable date to come back for data collection, and on the agreed date, the consent form was signed before data collection, which was conducted in a quiet environment to minimise disruptions.

### Data collection

Data were collected by the first author from October 2022 to January 2023 using individual in-depth interviews. Interview guides containing open-ended questions were used to guide the discussions and collect data from nurses and policymakers. To promote privacy, interviews were conducted in a quiet room allocated to the researcher. For HCPs, the broad opening question was, ‘What are your experiences with HIV self-disclosure in adolescents born with HIV?’ Interviews were conducted in both English and siSwati, and it lasted approximately 30 min. Follow-up questions enquired about: steps followed in preparing adolescents for self-disclosure, how adolescents can be empowered to self-disclose to others and the availability of training in assisting ALHIV to disclose? For policymakers, interviews lasting 45 min were conducted in English and questions asked focused on: availability of funds to facilitate HIV prevention programmes, provision of refresher courses to nurses for them to provide comprehensive services to ALHIV and development of national self-disclosure guidelines for ALHIV.

In Eswatini, there are two official languages: English and siSwati. English is mainly used as a business language while siSwati is used to communicate among native Swatis and is widely spoken by approximately 95% of Swati people. The interview guide was translated into siSwati and back translated to English to ensure accuracy. Demographic details of all participants were also collected.

### Data analysis

All the interview recordings were transcribed verbatim and those interview recordings in siSwati were then translated into English. Data were analysed using open, axial and selective coding, as proposed by Corbin and Strauss.^14^ In the open coding stage, the interview transcripts were repeatedly read until the researcher got a sense of the data. The transcripts were read line by line, and key phrases were identified and highlighted to uncover the participants’ thoughts, ideas and meanings.^14^ Thereafter, concepts that were similar or related in meaning were grouped together into subcategories and categories. Emergent categories were then closely examined and compared for similarities and differences. In the axial coding stage, the categories were formed by relating them to their subcategories to form more precise and complete explanations.^14^ Throughout the data-analysis process, there was reflection on the emerging findings to derive meaning from the data. Finally, selective coding was carried out, core categories were identified and coding was limited to those subcategories that relate to the core category in significant ways. Informed by the data, a focused literature review was conducted comparing the emerging concepts from the data with the existing studies and theoretical frameworks in order to complete the emerging theory, leading to the refinement of categories and subcategories.^14^

### Study rigour

The scientific rigour of the study was ensured by utilising Lincoln and Guba’s trustworthiness criteria. Credibility was ensured through the researcher spending some time with the participants before data collection to gain their trust so that they could provide rich data.^19^ Semi-structured interview guides also permitted flexibility during the interviews to allow the researcher to probe deeper as required.^20^ Dependability refers to the stability of the data over time and over the conditions of the study.^21^ It was ensured by using appropriate data collection instruments to elicit in-depth responses relevant to the research question and keeping a detailed track record of the entire data collection process.^19^ Furthermore, an audit trail was established whereby a detailed track record of the data collection process was developed, and during data analysis, the transcripts were reread against the audio files for accuracy. Confirmability was ensured by tape-recording data collection, writing field notes and a reflective journal, which was kept throughout the data collection process to avoid researcher bias. The reflective journal also assisted in capturing relevant and useful information, including participants’ quotations to validate the findings that are provided in the transcripts and the final step of producing a research report.^19^

Transferability, which is the extent to which results can be generalised to other contexts, was ensured by utilising purposive sampling techniques to make sure that they were appropriately suited to answer the research questions and that they were a true representation of the population under study. The provision of a detailed description of the number of participants who met the inclusion criteria and were part of the study, using the appropriate data collection methods, and stating the number and length of the data collection sessions including the time period over which the data were collected also assured transferability.^20^ Data were also collected until saturation was reached, which was evident when no new information emerged. The quality of information gathered from participants took precedence to determine the final size of the sample.

### Ethical Considerations

The study was conducted after obtaining ethical clearance from the University of KwaZulu-Natal Higher Degrees Ethics Committee (BREC/00002527/2021) and the Eswatini Health & Human Research Review Board (EHHRRB064/2021), as well as permission from the respective facilities where data was collected. Participation in this study was voluntary, and participants were free to withdraw at any time. Confidentiality, privacy and anonymity were respected during the data collection process. All participants signed consent forms before data collection. Demographic forms did not bear the names of participants, but instead, codes were used to protect their identity and were thereafter kept safe in a lockable cabinet in the researcher’s office.

## Results

### Demographic characteristics of participants

A total of 28 participants took part in the study. A majority of the nurses were female, 16 (67%), while 8 (33%) were male. For policymakers, 3 (75%) were females, while 1 (25%) was male. About half of the nurses, 12 (50%) fell within the age bracket of 25–30 years, 9 (37.5%) were in the age bracket 31–40 and 3 (12.5%) were in the 41–50 age bracket. About half 12 (50%) of nurses had worked in the ART clinic for 2–3 years, another 10 (42%) had 4–5 years of experience in the ART clinic, while only 2 (8%) had 6 years of experience. In-service disclosure training was provided to 7 (29%) nurses and 17 (71%) had not partaken in in-service disclosure training. All policymakers had served more than 3 years in their current positions. Demographic details of participants are outlined in Table 1.

**TABLE 1:** Nurses and policymakers demographic characteristics.

Characteristics	*n*	%
**Nurses**		
**Gender**		
Female	16	67.0
Male	8	33.0
**Age (years)**		
25–30	12	50.0
31–40	9	37.5
41–50	3	12.5
**Number of years working in ART clinic**		
2–3	12	50.0
4–5	10	42.0
6 <	2	8.0
**Received in-service disclosure training**		
Yes	7	29.0
No	17	71.0
**Policymakers**		
**Gender**		
Female	3	75.0
Male	1	25.0
**Age (years)**		
31–40	1	25.0
41–50	3	75.0
**Number of years in current position**		
3 <	4	100.0

ART, anti-retroviral therapy.

Data analysis revealed five categories, which included: (1) comprehensive education to ALHIV, (2) teen club affiliation, (3) adolescent-friendly services, (4) right to self-determination and (5) barriers in facilitating self-disclosure. Table 2 summarises the categories and subcategories of the findings.

**TABLE 2:** Categories and subcategories.

Categories	Subcategories
Comprehensive education to ALHIV	Empowering adolescents with basic HIV information and benefits of disclosure Pre-disclosure assessment
Teen club affiliation	Regular attendance of teen club meetings Utilising peer educators Ongoing psychosocial support
Adolescent friendly services	Adolescent friendly corner
Right to self-determination	Disclosure readiness Consequences of disclosure
Barriers to facilitating self-disclosure	Nurses lack of training Unavailability of self-disclosure guidelines

ALHIV, adolescents living with HIV.

### Comprehensive education for adolescents living with HIV

Participants in this study asserted that for adolescents to disclose, they required adequate information, and this, therefore, entailed empowering them with basic HIV information and benefits of disclosure and adolescent assessment of the person to disclose to.

### Empowering adolescents with basic HIV information and benefits of disclosure

In preparing adolescents for HIV status disclosure, data revealed that nurses empowered adolescents with HIV information all over again despite that it was previously given to adolescents. This was to help refresh their memory and to equip them. This included information such as what is HIV, how it is transmitted, how to prevent transmission, how to live healthy with HIV, taking treatment, and rights and responsibilities that come with living with HIV. This is what some participants had to say:

‘The first step is to reinforce the information that you have given to the adolescent about HIV before, in order to ensure they have the right information.’ (P14, nurse, IDI)

‘Information is power, so adolescents need to be empowered with adequate information before they can be in a position to disclose.’ (P5, nurse, IDI)

Participants stated that adolescents are also informed of the benefits of disclosure and how disclosing assists them in getting social support from the person they are disclosing to, enhances their self-esteem and promotes trust in relationships. However, adolescents should utilise ‘why’, ‘when’ and ‘how’, so that they understand why an individual should disclose, when to disclose and how to disclose. The following statements confirm this:

‘Disclosure is good for the person who is disclosing; it gives them that feeling of doing something right.’ (P2, nurse, IDI)

‘We tell them that after you have disclosed, the individual will take the role of supporting you in that situation.’ (P16, nurse, IDI)

### Pre-disclosure assessment

Some participants also explained that it is crucial for adolescents to assess the HIV knowledge of the person they plan to disclose to by asking questions, and if they felt there was a gap in HIV knowledge, then they would educate that person first with the right information and then progressively move towards disclosure of their status. This is what some participants said:

‘Adolescents are told the importance of first assessing what the person they want to disclose to knows about HIV, if it is clear they have sufficient information about HIV, then disclosure can happen.’ (P9, nurse, IDI)

‘Disclosing to someone who has very little knowledge about HIV may lead to rejection, so it is not advisable to disclose if someone is not well informed.’ (P6, nurse, IDI)

### Teen club affiliation

Adolescents do not inherently have self-disclosure skills and regular attendance of teen club meetings, utilising peer educators and ongoing psychosocial support would help prepare them for disclosure.

### Regular attendance of teen club meetings

Participants stated that they encouraged ALHIV to meet with other adolescents in the teen club because it ensured age-specific peer support for adolescents and made it easier to educate them about issues that confront them. During the teen club meetings, adolescents are grouped according to their ages as their levels of understanding are different, and this helps to focus mainly on the issues that are relevant to them. The younger adolescents aged 10–14 years are taught about treatment adherence and living a healthy lifestyle and the mature 15–19-year-olds are given additional information such as HIV prevention and transmission, how to use condoms and self-disclosure. The following excerpts confirm this:

‘Here we have the teen club, and the children come on Saturday once a month. This is a good place to see them as a group and teach them in general because others get the message better when it’s like a relaxed and jolly environment than having a serious setup.’ (P3, nurse, IDI).

‘Talking to them is okay, and then introduce them to teen clubs because that is where there are other children, and they can relate well since they are all on treatment.’ (P4, nurse, IDI).

‘Topics usually covered are sexual education, how to use protection, importance of adherence.’ (P7, nurse, IDI)

### Utilising peer educators

Participants stated that community adolescent treatment supporters (CATS) peer educators were also invited during teen club meetings to come and motivate the adolescents. The training of the CATS is carried out at the national level, and they are then attached to facilities to assist ALHIV who may have challenges. Adolescents communicate better with their peers and are able to ask questions they would otherwise not ask. Furthermore, other adolescents who have already disclosed are invited to come and share their experiences as a way of motivating others to also disclose their status. This is what the participants had to say:

‘In these meetings they are free to discuss and state their fears around disclosure like being rejected and stigmatised after disclosure and concerns about the confidentiality of their status because they are peers.’ (P18, nurse, IDI).

‘Bringing those who have already disclosed their status benefits the adolescents because they can see that it can be done.’ (P20, nurse, IDI)

### Ongoing psychosocial support

Most participants explained that in order to self-disclose, ALHIV need ongoing support, initially after they have been disclosed to and thereafter, to assist them in coping with the physical and mental difficulties of living with HIV and to make it possible for them to disclose their status to others:

‘I think we need psychological support, especially for the children, ongoing counselling because another child may say they are okay whereas they are not’ (P5, nurse, IDI)

‘The children need continuous counselling because they have many issues to deal with like anger, and it’s the anger that makes them not care who they also infect.’ (P9, nurse, IDI).

### Adolescent friendly services

Participants explained that adolescents should be acknowledged as a special population with their unique needs, and this required an adolescent-friendly corner in facilities.

### Adolescent friendly corner

To serve adolescents effectively, there should be the availability of services that cater for adolescents specifically. This requires having personnel who are willing to work with adolescents who should be trained on how to best deal with adolescents. Having a friendly corner acts as a safe space for adolescents to talk about what bothers them and makes it possible to have one-on-one sessions for adolescents who require it. The excerpt below confirms this:

‘There is a need to ensure that the adolescent-friendly corners in facilities are active as they promote the delivery of services in a way that adolescents regard as good.’ (P3, nurse, IDI)

Participants stated that adolescents also utilise a drop-in box where they write suggestions on how they would like their services to be rendered, and they also use the drop-in box to write on whatever topics they wish to be addressed, and then those topics are discussed during teen club meetings. The following statements confirm this:

‘Adolescents are at most times discreet, and they prefer to write their thoughts and use the drop-in box than to say some things face to face, that is not easy for them.’ (P14, nurse, IDI)

‘The drop-in box has made it possible for us to know exactly what the adolescents want, and this has helped us to improve our services for them.’ (P5, nurse, IDI)

### Right to self-determination

After being prepared to disclose, adolescents should make the final decision on when and to whom they disclose to, but that requires being ready first to disclose and being aware that there will be consequences after disclosure.

### Disclosure readiness

Participants also emphasised that preparing adolescents to disclose did not take away their autonomy to decide when to disclose because disclosure was an individual decision that can never be imposed upon anyone, and they should be allowed to go ahead with disclosure to the person they have chosen once they feel ready to do so. The following excerpts confirm this:

‘After having been given all the information, they should make the decision whether to disclose or not, to whom or when, is their individual decision’ (P23, nurse, IDI)

‘ALHIV are encouraged to disclose, but they have the final say to do it when they want to.’ (P16, nurse, IDI)

### Consequences of disclosure

Disclosure has been known to come with numerous reactions, and therefore adolescents need to understand, before they disclose, that the reactions after disclosure may either be negative or positive. Participants declared that adolescents needed a strong identity of self and should be ready to assert themselves despite having a positive HIV status as this would help them to cope with the consequences of disclosure better:

‘We need to empower them such that even if they come across the stigma and discrimination after disclosure, they are aware of who they are, this would boost their self-esteem and confidence and help them to accept themselves.’ (P6, nurse, IDI).

‘Our adolescents need to have a mindset that this is me, and you cannot change me. I’m a young person, and living with HIV is just another piece of the puzzle, but I have goals to achieve. If one piece fell on the floor, it’s okay I still have other pieces, so let not the one that has fallen down come with sand and spoil everything.’ (P27, policymaker, IDI)

‘So we also empower them to say, after you have disclosed, how do you then cope because with disclosure it’s like now that I have told the person, I wonder what he and/or she is thinking about me.’ (P27, policymaker, IDI).

‘They need to be educated more, to eventually realise the advantage of disclosure, how self-disclosure would help them no matter how people react thereafter.’ (P11, nurse, IDI).

Some participants stated that adolescents should be empowered to take the step of going back to the person they have disclosed to in order to find out how they are feeling after being told about the status because it would help to give the individual a chance to ask questions and share their thoughts about the disclosure:

‘We are trying to educate them to understand that you also need to take part and make sure that after you have disclosed, you also do a follow-up to check: how do you feel after I have told you about my status? What is your thinking? What are your fears”?’ (P10, nurse, IDI)

‘Disclosure may shock the other person, and adolescents have to realise their responsibility after disclosure, that is reaching out to the disclosed person.’ (P4, nurse, IDI)

### Barriers to facilitating self-disclosure

Participants in this study opined that there were barriers that were hindering adolescent self-disclosure, which were not directly related to the adolescents but were service delivery related.

### A lack of training for nurses

A lack of training made it difficult for nurses to effectively prepare adolescents to disclose because they lacked the appropriate skills. Most participants did not receive any formal training on disclosure counselling for children but rather relied on experience. The on-the-job training that they received was not sufficient, and it left them feeling inadequate to appropriately execute their duties. A youth-friendly training was deemed necessary on top of the disclosure training because adolescents were a special population with their own unique needs and special language that nurses needed to understand if they were to communicate effectively with them:

‘We should also be empowered in terms of handling the adolescents themselves; be adolescent-friendly because you don’t handle adolescents the same as adults. Those trainings are not being done.’ (P18, nurse, IDI)

Adolescent HIV management workshops and in-service education were not routinely performed because such training mostly relied on funders, and this made it difficult to train the number of nurses that would meet the current demand for their services. Incorporating adolescent health in the pre-service curriculum would, however, go a long way in bridging the gap that has been identified:

‘So most of the time, the funding limits us. If only we had so much funding that at least per year you are allowed to train at least 300 so that at least per quarter we train a certain number.’ (P26, policymaker, IDI).

‘In fact I wish our nursing schools could incorporate adolescent health in their curriculum so that we could have such things covered.’ (P26, policymaker, IDI).

### Unavailability of self-disclosure guidelines

Participants asserted that for adolescent self-disclosure to be implemented, there was a need to develop formal and standardised disclosure guidelines. These guidelines should be developed at the national level and then cascaded down to facilities so that every nurse can access them, consequently resulting in improved adolescent self-disclosure rates. This is what some participants articulated:

‘We don’t basically have a disclosure guideline or let me say I haven’t seen any which we use’ (P1, nurse, IDI)

‘I think when we look at self-disclosure guidelines; we don’t have such a document. We need to have it; there is a gap’ (P25, policymaker, IDI).

## Discussion

This study examined the process followed by nurses in preparing adolescents for self-disclosure and explored the role of policymakers in enabling adolescents to disclose their HIV status to others. Participants in our study stated that the initial step in preparing adolescents was to provide comprehensive HIV education to equip them for the disclosure event, which could be met with questions from the person they were disclosing to. If the adolescent has enough information, they will be able to respond appropriately when asked questions. However, before the disclosure, adolescents should firstly ascertain how much the person knows in order to educate that individual before disclosure. These findings resonate with findings from a study conducted by Hogwood et al., where participants stated that society lacked sufficient knowledge about HIV, especially their peers, and further mentioned that more education was needed to raise awareness.^22^

Secondly, participants in the study also mentioned that they made adolescents aware of the benefits they would get from self-disclosure, which was social support and enhanced self-esteem, both of which are important for the ALHIV. The disclosure processes model (DPM) provides a theoretical framework to understand when and why interpersonal, verbal self-disclosure is beneficial for individuals who live with concealable stigmatised identities such as HIV.^23^ When applied to ALHIV, the DPM would seek to answer when disclosure would be beneficial instead of harmful. The model asserts that a positive reaction of the confidant to the disclosure plays a key role in predicting whether disclosure will be regarded as beneficial or not.^24^ The model further posits that it is also necessary to understand why disclosure affects well-being and proposes that disclosure of the HIV status will bring about positive psychological, behavioural and health outcomes.^24^

Participants in our study emphasised that enrolling adolescents in the teen club was another way of preparing adolescents for self-disclosure. This was because the teen club created a conducive environment for peer-to-peer support, including education about specific topics that were easier to tackle in a group setup. Findings were that peer-to-peer support gained during the teen club also helped the adolescents to talk to each other openly in order to come up with concrete solutions to challenges they are facing, and they concur with a study conducted in Ghana by Barker et al. and another study conducted in South Africa by Rencken et al. that being in a teen club fosters open communication, honesty and trust among ALHIV and the difficulties they experience adhering to treatment and accepting their HIV status.^25,26^

Participants also stated that ALHIV need on-going support to improve their physical, emotional and overall wellbeing. A previous study by Atuyambe et al. agrees with these findings that psychosocial support will encourage access to ART services and adherence to treatment for people living with HIV, thus achieving an optimal state of physical health, which acts as an influence on emotional and mental well-being.^27,28^ This would be valuable as ALHIV have in the past expressed a strong need for support around the formidable time of adolescence.^9^ The study also revealed that adolescent-friendly services have to be provided to enable adolescents to communicate better with HCPs and, therefore, have their specific needs met. This calls for these services to be active and strengthened for them to be effective and for nurses to understand that they need to change their attitudes and accommodate ALHIV.

Some participants in our study emphasised that after adolescents have been prepared for self-disclosure, they should not be coerced to disclose but be allowed to disclose at their own time when they have satisfied themselves that they are ready to disclose as the disclosure was an individual decision. These findings concur with a previous study conducted in Zimbabwe by Mlilo et al. that HIV status disclosure is a personal decision that adolescents undertake to assert their autonomy.^29,30^ Participants in the study also empowered the ALHIV on how to deal with possible negative reactions after they have disclosed their status and this empowerment includes building the self-esteem of adolescents, which leads to adolescents having a strong sense of self-assurance despite facing possible stigmatisation and discrimination.

More than half of the nurses in our study considered themselves not to have adequate knowledge about how to help adolescents disclose. The in-service training they got was not enough, leaving them feeling incompetent. As adolescents have special needs peculiar to them, there is a critical need to train healthcare workers, which will focus on counselling young people.^31^ Training is not the only requirement for effective disclosure, but the lack of standardised disclosure guidelines also contributes to discrepancies in nurses’ implementation of the disclosure.^10^ The lack of formal self-disclosure guidelines was reported as one of the key concerns by nurses, hindering adequate preparation of adolescents to self-disclose. It is important to attend to the current gap around adolescent HIV disclosure training and the actual practice by nurses so as to improve their performance.^32^ Culturally tailored interventions for ALHIV must be developed and meticulously appraised to determine if they meet this unique population’s needs.^33^

### Strengths and limitations of the study

The strengths of the study are that: (1) it drew on the perspectives of nurses and policymakers to provide insight into measures that are utilised to prepare adolescents for self-disclosure, (2) it further provides evidence that adolescent HIV self-disclosure has not been extensively scrutinised to better understand how it can be improved and (3) because of the nature of the study being qualitative, the study also explored participants’ thoughts about the phenomenon under study to gain a deeper insight. Furthermore, this study has the unique contribution of being the first study in Eswatini that has interrogated the process followed by nurses in preparing ALHIV to disclose as well as the role played by policymakers in enabling ALHIV to disclose. The study limitations are: (1) the study did not explore how nurses can collaborate with caregivers of ALHIV in preparing adolescents to disclose and (2) the study population is not representative of all nurses caring for ALHIV. Furthermore, future studies with a larger sample are necessary for a holistic understanding of the role of nurses and policymakers in preparing adolescents for status self-disclosure.

## Conclusion and recommendations

This study explored the role of nurses and policymakers in facilitating and promoting adolescent self-disclosure. The main findings from the study are that ALHIV are not being adequately prepared to disclose. Nurses are not trained in facilitating self-disclosure and, therefore, are not preparing adolescents satisfactorily to disclose. Furthermore, the non-availability of HIV self-disclosure guidelines also hinders disclosure. The development of standardised disclosure guidelines would give nurses better means to support ALHIV with the self-disclosure process. Policymakers’ lack of funding for the training of nurses involved in adolescent HIV management also compounds this already existing challenge. We presume that prioritising the training of nurses and developing self-disclosure guidelines would lead to a remarkable increase in the rate of HIV self-disclosure by Swati adolescents.
